# Effect of the inhaled anesthetics isoflurane, sevoflurane and desflurane on the neuropathogenesis of Alzheimer’s disease (Review)

**DOI:** 10.3892/mmr.2015.3424

**Published:** 2015-03-04

**Authors:** JUE JIANG, HONG JIANG

**Affiliations:** Department of Anesthesiology, Shanghai Ninth People’s Hospital, Shanghai Jiao Tong University School of Medicine, Shanghai 200011, P.R. China

**Keywords:** Alzheimer’s disease, isoflurane, sevoflurane, desflurane, β-amyloid protein, tau, cognitive deficits

## Abstract

The incidence of Alzheimer’s disease (AD) in individuals >65 years of age is 13% and ~66 million individuals in this age group undergo surgery annually under anesthesia. It is therefore important to determine whether commonly used inhaled anesthetics induce cytotoxicity, which may lead to neurodegeneration. Findings from several studies suggest that the anesthetics, isoflurane, sevoflurane and desflurane, may activate caspases, increase the synthesis and accumulation of β-amyloid (Aβ) protein, and induce hyperphosphorylation of tau proteins, all of which are cellular responses consistent with the neuropathogenesis of AD. Other studies have arrived at different and occasionally contradictory conclusions. The present review attempts to resolve this discrepancy by reviewing previous studies, which have investigated the effects of commonly used inhaled anesthetics on the synthesis and accumulation of Aβ, tau pathology and cognitive function. The possible underlying mechanism was also reviewed. However, several aspects of this phenomenon remain to be elucidated. Further studies are required to fully examine anesthesia-induced neurotoxicity and elucidate the effect of inhaled anesthetics on the onset and progression of AD.

## 1. Introduction

Alzheimer’s disease (AD) is a multi-factorial and heterogeneous neurodegenerative disorder. It is irreversible, insidious and characterized by progressive worsening of symptoms, including a global cognitive decline in memory, orientation, judgment and reasoning. The cellular etiology of AD is associated with the loss of neurons and synapses in cortical and limbic structures, including the hippocampus and amygdala. Currently, AD has a incidence of 13% in individuals >65 years of age (1); however, its prevalence is expected to quadruple by 2050. Without a significant therapeutic breakthrough, 1/85 individuals worldwide are likely be living with this disease (2). Furthermore, the demographics of patients undergoing surgery under the influence of anesthesia annually indicates that ~66 million individuals are >65 years old (1). As life expectancy continues to increase, the number of patients with AD requiring surgery and administration of anesthesia may also steadily rise. Studies have demonstrated that inhaled anesthetics, including isoflurane, sevoflurane and desflurane, have an impact on the neuropathogenesis of AD and possibly accelerate the clinical progression of this neurodegenerative disorder (3,4). Other previous studies have demonstrated that the administration of general anesthesia may be a risk factor for the development of AD (5–9). In addition, the expression levels of tau protein and certain cytokines in the cerebrospinal fluid (CSF) following anesthesia and surgery are consistent with those identified in patients with AD (10). However, certain studies have arrived at different conclusions and have suggested that anesthesia and surgery may not contribute to the development of AD (11,12). Thus, it has been difficult to clinically prove the association between anesthesia and AD. Evidence from several previous studies on the impact of isoflurane, sevoflurane and desflurane on the neurotoxicity and neuropathogenesis of AD may assist in facilitating and guiding future clinical studies. In the present review, studies assessing the effects of commonly used inhaled anesthetics on the processing of amyloid precursor protein (APP), metabolism of β-amyloid protein (Aβ), tau pathology, synaptic plasticity and cognitive deficits are discussed. Furthermore, the molecular mechanisms underlying the anesthetic ally-induced development of an AD-like neuropathology are reviewed. Finally, the present review offers a perspective on future studies, which are required to shed further light on this field of study.

## 2. Neuropathogenesis of AD

### Neuropathogenic hallmarks of AD

**Figure 1:**
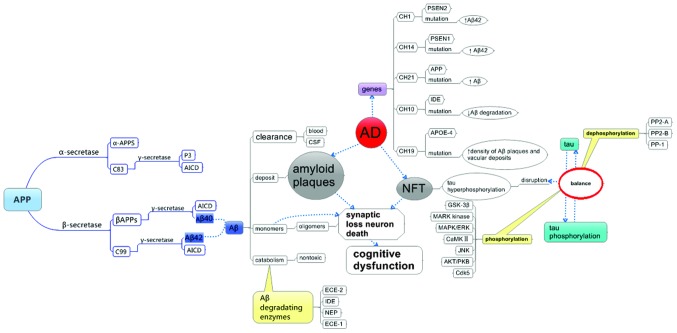
AD-associated genes, hallmarks of AD and its processing. AD, Alzheimer’s disease; APP, amyloid precursor protein; APPS, large soluble ectodomain of APP; AICD, β-amyloid precursor protein intracellular domain; Aβ; β-amyloid; CSF, cerebrospinal fluid; ECE, endothelin-converting enzyme; IDE, insulin degrading enzyme; NEP, neprilysin; NFT, neurofibrillary tangles; PSEN, presenilin; APOE, apolipoprotein E; GSK, glycogen synthase kinase; MARK, microtubule affinity regulating kinase; ERK, extracellular regulated kinase; MAPK, mitogen-activated protein kinase; CaMKII, calcium/calmodulin-depnedent kinase; JNK, c-Jun N-terminal kinases; PKB, protein kinase B; Cdk, cyclin-dependent kinase; PP, protein phosphatase.

The two most important histological features of AD are the formation of extracellular amyloid plaques, composed predominantly of Aβ, and intraneuronal neurofibrillary tangles (NFT), composed of aberrantly hyperphosphorylated tau proteins assembled into paired helical filaments (PHF) (13). Other characteristics of AD are dystrophic neurites, extensive neuronal loss and gliosis. Amyloid plaques are predominantly composed of Aβ40 and Aβ42, which are generated through the amyloidogenic processing of APP, requiring the activity of the enzymes β- and γ-secretase (14). Tau is a microtubule-associated protein, which is normally enriched in the axons. However, during AD and other tauopathies, it becomes hyperphosphorylated, and is subsequently relocalized to and aggregates in the somatodendritic compartment of the affected neurons. Hyperphosphorylated tau proteins assemble into PHF structures, which in turn induce the formation of NFTs (13,15,16). The final outcome of this pathological process, as shown in Fig. 1, is neuronal cell death and degeneration, as observed in AD (15).

### APP processing and Aβ metabolism

Aβ is the major component of the amyloid plaques observed in the brains of patients with AD. The imbalance between the generation and clearance of Aβ leads to its accumulation, one of the fundamental molecular features of AD contributing to its neuropathology.

Aβ is produced by the serial proteolysis of APP by enzymes, including α-secretase, aspartyl protease β-site APP-cleaving enzyme (BACE), β-secretase and α-secretase, as reviewed previously (17) and shown in Fig. 1. α-secretase cleaves APP at a site close to the transmembrane domain and in the middle of the Aβ region, to release a large soluble ectodomain (α-APPs) into the lumen/extracellular space, while retaining a C-terminal fragment of 83 amino acids (APP-C83) in the membrane. APP-C83 is further cleaved by α-secretase into p3, an amino-terminal truncated form of Aβ. By contrast, β-secretase cleaves APP to generate a 99-residue membrane-associated C-terminal fragment (APP-C99). This is subsequently cleaved by α-secretase to release a 4-kDa Aβ protein and the β-amyloid precursor protein intracellular domain. This cleavage by α-secretase is an unusual form of proteolysis as the protein is cleaved within the transmembrane domain (at residue +40 or +42) (18–20). APP also undergoes caspase-mediated cleavage to generate a 90-kDa N-terminal APP-caspase fragment. The cleavage of APP into the toxic Aβ peptides results from the activity of β-secretase. Therefore, increased proteolysis by α-secretase may alter the balance and lead to decreased production of Aβ.

Aβ is cleared from the extracellular space and moved into the blood and CSF, and it can also be degraded into less neurotoxic metabolites (21). The enzymes involved in Aβ turnover include, insulin degrading enzyme (IDE), neprilysin (NEP) (21–25), endothelin-converting enzyme (ECE)-1, ECE-2 and possibly plasmin, as previously described (21,23). NEP, known to be important in Aβ catabolism, is a 90–110 kDa plasma membrane glycoprotein of the neutral zinc metalloendopeptidase family (26,27) and has a higher tendency to cleave Aβ40 than Aβ42 (28). The activity of NEP is regulated by factors that affect the onset of AD, including aging, estrogen levels, exercise and environmental enrichment. IDE, a zinc metalloendopeptidase, is another protease important in regulating the levels of Aβ in the brain. Certain risk factors associated with AD, including diabetes mellitus, hyperinsulinemia and apolipoprotein E (APOE)-ε4 allele, may facilitate disease onset, at least partly, by affecting the activity of IDE (29–33). ECE-1 and ECE-2 contribute to the regulation of steady-state Aβ levels in the brain (23).

Aβ oligomers are formed through the aggregation of the less toxic Aβ monomers. They lead to synaptic dysfunction and neuronal damage (34,35), and have been extensively reviewed (35). Previous studies have demonstrated a robust correlation between the levels of soluble Aβ oligomers and the extent of synaptic loss, and the severity of cognitive impairment (36–41). This suggests that inhibition of Aβ oligomerization may be a promising approach for the prevention and treatment of AD (34,35).

### Tau pathogenesis

In the normal brain, localization of tau is restricted to the axonal compartment of neurons (42). Tau proteins are members of a family of microtubule-associated proteins, which are important in the assembly of microtubules, contribute to axonal integrity in mature neurons (43,44), and perform functions at the dendritic and nuclear level in neurons (45,46). Hyperphosphorylated and abnormally phosphorylated forms of tau are the major constituents of the intraneuronal PHFs observed in AD. They are also detected in similar filaments observed in other neurodegenerative disorders, termed tauopathies (43,47,48). Spatiotemporal progression of tau aggregates from the entorhinal cortex and hippocampus to isocortical areas (49) has been correlated with cognitive deficits (50), and accumulation of hyperphosphorylated tau is associated with memory impairment in several animal models (8,51–53). These findings support the pivotal role of tau pathology in AD-associated memory loss.

Homeostasis of tau phosphorylation is maintained through a balance in the activity of enzymes, which catalyze the phosphorylation and the dephosphorylation of tau, as shown in Fig. 1. Tau phosphorylation is mediated by kinases, including glycogen synthase kinase-3β (GSK-3β), MARK kinase, mitogen-activated protein kinase/extracellular signal-regulated kinase (MAPK/ERK), calcium/calmodulin-dependent protein kinase II, c-Jun N-terminal kinase, protein kinase B (AKT/PKB) and cyclin-dependent kinase 5 (Cdk5), which includes the catalytic Cdk5 and the p35, p25 and p39 regulatory proteins (54,55). By contrast, dephosphorylation is mediated by protein phosphatase (PP)-2A, PP-2B and PP-1. PP-2A is the most important phosphatase accounting for >70% of tau dephosphorylation in the brain and its activity is downregulated in AD (15). Disruption in the homeostasis of tau phosphorylation results from a dysregulation of tau-associated kinases and phosphatases, eventually leading to the formation of neurofibrillary tangles and causing the neuronal cell death exhibited in AD (15). At the functional level, hyperphosphorylation of tau impairs its microtubule-binding properties, resulting in its detachment. This causes destabilization of microtubules, disrupts axonal transport and eventually leads to the relocalization of tau to the somatodendritic compartment where NFTs have been identified in AD and other tauopathies (56–58). The appearance of tau aggregates is correlated with a loss of microtubules and the breakdown of normal axonal transport (59). Tau pathology also correlates with the onset and progression of dementia in AD, and memory loss and mild cognitive impairment during aging (60).

### Aβ, tau and AD

Small and Duff (61) suggested two hypotheses, which may explain Aβ and tau causality, termed the ‘dual pathway model’ and ‘serial model’ (62,63). According to the dual pathway model, an insult may induce an increase in the production of Aβ and the phosphorylation of tau simultaneously, which then independently leads to synaptic loss and dementia (61). A series of genetic and experimental findings form the basis of the ‘amyloid hypothesis’, which suggests a serial model of causality. According to this hypothesis, increased production and deposition of Aβ is important in triggering neuronal dysfunction and death in AD (62). Therefore, an increase in Aβ is the prime pathogenic driver, which in turn leads to the hyperphosphorylation of tau and other histological and clinical symptoms of AD, including synaptic loss and dementia. A previous study demonstrated that soluble Aβ dimers isolated from the cortex of patients with AD, directly induces the phosphorylation of tau and neuritic degeneration (64). This study demonstrated that subnanomolar concentrations of cortical Aβ dimers from patients with AD, the most abundant form of soluble oligomers detectable in the human brain, first induced hyperphosphorylation of tau at AD-relevant epitopes in hippocampal neurons, subsequently disrupting the microtubule cytoskeleton and causing degeneration of neuritis (64). Purified, synthetic dimers exerted identical effects to the natural AD dimers (64). It was also revealed that knocking down the expression of endogenous tau fully prevented neuritic changes and by contrast, overexpression of human tau accelerated these changes (64). Co-administration of antibodies recognizing the Aβ N-terminus prevented cytoskeletal disruption (64). Based on these findings, it was suggested that natural Aβ dimers isolated from the brain of patients with AD are sufficient to induce AD-type hyperphosphorylation of tau, followed by neuritic dystrophy. However, passive immunotherapy mitigates this effect (64). Other previous studies have demonstrated that Aβ oligomers are also capable of inducing the phosphorylation of tau (65). Previous studies in mice have provided evidence to support a model of the pathogenesis of AD, in which soluble Aβ oligomers trigger synaptic dysfunction; however, the formation of abnormal tau aggregates is required to induce neuronal death severe enough to result in cognitive decline and dementia. This has been reviewed previously (34). At the physiological level, it is known that while accumulation of intraneuronal Aβ causes deficits in long-term synaptic plasticity, synaptic dysfunction and long-term potentiation deficits manifest in an age-associated manner prior to the appearance of plaques and tangles (66).

## 3. AD-associated genes

The genes encoding APP, presenilin (PSEN)-1 and PSEN-2, have been demonstrated to harbor autosomal dominant mutations associated with AD, while the presence of the APOE-4 allele is considered a risk factor for the development of AD (Table I) (67). APP and PSEN mutations lead to increased production of Aβ42 peptides, while inheritance of ApoE4 alleles cause an increase in the steady-state levels of Aβ in the brain, as reviewed previously (14). PSEN-1 mutations, therefore, render neurons vulnerable to isoflurane toxicity (68). The 3 × Tg-AD mouse model exhibits mutations in three AD-associated genes, human β-amyloid precursor protein (APPSwe), PSEN-1 (PS-1M146V) and tau (tauP301L), and develops Aβ plaques, NFTs and exhibits cognitive impairment (66,69). Liang *et al* (68) revealed that neurons exhibiting one presenilin-1 mutation were susceptible to isoflurane-induced cytotoxicity and increased cytosolic calcium levels. Certain studies have suggested that the transgenic mouse models of AD may be more susceptible to developing neurotoxicity compared with the wild-type mice following the administration of isoflurane (70) and sevoflurane (71). These findings suggest that patients exhibiting AD-associated gene mutations may be at an increased risk of developing anesthesia-induced neurotoxicity. Further evidence supporting the genetic component of AD etiology comes from investigations, which correlated genomic variations in close proximity to the IDE gene with disease severity, plaque and NFT density (72), and the plasma levels of Aβ42 in patients with AD (73).

## 4. Effects of inhaled anesthetics on Aβ

### Isoflurane

An *in vitro* study demonstrated that isoflurane promotes the oligomerization of Aβ and increases its toxicity (5). A combination of inhaled anesthetics and hypoxia may activate caspases and induce apoptosis, increasing the overall level of amyloid proteins (3,74). Xie *et al* (3) reported that exposure to 2% isoflurane for 6 h induces apoptosis, alters the processing of APP and leads to an increased production of Aβ peptides in H4 human neuroglioma cells stably transfected to express human wild-type full-length APP (H4-APP cells). Isoflurane also increases the rate of Aβ oligomerization and pheochromocytoma cytotoxicity *in vitro* (5,75) by exhibiting a preference for binding small oligomeric species (5). Repetitive exposure to 2% isoflurane (twice weekly for 3 months) increased the quantity of Aβ aggregates in APP mice compared with the wild-type (70). A clinically relevant form of isoflurane anesthetic (1.4% isoflurane for 2 h) was revealed to induce the activation of caspases with modest increases in the levels of BACE and Aβ in the mouse brain between 6 and 24 h following administration (76) In humans, isoflurane induces an increase in the levels of Aβ40 in the CSF 24 h following surgery under the influence of the anesthetic (77). Previous studies have achieved success in mitigating these effects. For example, the caspase inhibitor, Z-VAD, has been demonstrated to attenuate isoflurane-induced caspase activation, APP processing, Aβ accumulation and apoptosis in H4-APP cells (74). Inhibitors of Aβ aggregation, iAβ5 and clioquinol, selectively attenuate the isoflurane-induced activation of caspase-3 (74,76). However, in naïve H4 cells (not overexpressing APP), isoflurane induces the activation of caspase-3 in the absence of any detectable alterations in the generation of Aβ, although the latter may potentiate the activation of caspases (74). These findings suggest that the caspases activated by isoflurane may in turn increase the activity of BACE, alter APP processing and increase the levels of Aβ to trigger further apoptosis (74,76). The result is a vicious cycle of anesthetic-induced apoptosis, generation and aggregation of Aβ leading to additional rounds of apoptosis, and eventually, debilitating levels of neurodegeneration. This conclusion is also supported by previous findings where a reduction in the levels of BACE and Aβ were demonstrated to attenuate the isoflurane-induced activation of caspase (78). Finally, treatment of H4-APP cells with a combination (however, not independent exposure) of 70% nitrous oxide and 1% isoflurane for 6 h induced the activation of caspase-3 and apoptosis, and increased the levels of BACE and Aβ peptides (79).

Notably, certain previous studies have failed to determine an association between exposure to anesthetic during 1–5 years preceding disease onset and the risk of developing AD (11). In behavioral assays, the performance of 85Dbo/J transgenic AD mice (APPswe, PSEN1dE9) and wild-type mice in the Morris Water Maze (MWM) test, improved significantly 48 h following 5-day exposure to isoflurane (80). The transgenic AD mice made significantly fewer discrimination errors in the Y maze following isoflurane administration and no differences were observed compared with the wild-type, until 5 months following exposure (80). During this period, the quantity of Aβ plaques and oligomers in the hippocampus were reduced significantly in the transgenic AD mice (80). These findings suggest that repeated isoflurane exposure during the pre-symptomatic phase improved spatial memory in the APP/PS1 transgenic and wild-type mice shortly following exposure, prevented an age-associated decline in learning and memory, and attenuated the formation of Aβ plaques and oligomers in the hippocampus (80).

### Sevoflurane

Sevoflurane induces identical cellular and histological effects to isoflurane. Exposure to 4.1% sevoflurane for 6 h induces apoptosis, alters APP processing and increases the production of Aβ in H4-APP human neuroglioma cells. This effect is attenuated by treatment with Z-VAD and the γ-secretase inhibitor, L-685,458, and is potentiated by Aβ (81). Previous *in vivo* studies exposed naïve mice to 2.5% sevoflurane for 2 h and observed increased levels of activated caspases, BACE and Aβ aggregates in the brain at 6, 12, and 24 h following anesthesia (76). Also, a combination of 2.1–3% sevoflurane and 60% oxygen for either 2 or 6 h successfully induced caspase activation and apoptosis, altered APP processing, and increased the levels of Aβ in the brains of 6-day-old mice (71). Therefore, sevoflurane appears to act through a vicious cycle similar to isoflurane, by triggering a cascade of caspase activation, increasing BACE activity, aberrant APP processing and increasing the generation and aggregation of Aβ, leading to further apoptosis (81).

By contrast, certain studies have reported that sevoflurane may either have no deleterious effect or, in certain cases, a neuroprotective function. For instance, a 4 h exposure to one minimum alveolar concentration of sevoflurane revealed no impairment in learning or memory, in young, adult and aged rats, according to the MWM test (82). Exposure 2.1% sevoflurane 4- and 16-times and exposure to 3% sevoflurane 16-times selectively rescued the Δelectroretinograms in (ΔERG) AD-transgenic flies, in which the ΔERG, climbing ability and survival rate were lowered; however, no affect was observed in control flies (83). These findings led to the conclusion that sevoflurane exerts no neurotoxic effects on AD-transgenic flies, however, may confer selective neuroprotection on their retinal function (83). Contradictory results, including these, warrant further studies to elucidate the effects of sevoflurane on AD-associated neurotoxicity and subsequent neuropathology (83).

### Desflurane

Desflurane is another commonly used inhaled anesthetic, which in contrast to isoflurane and sevoflurane, when supplied to H4-APP cells at a clinically relevant concentration (12%) for 6 h, revealed no induction in the activation of caspase-3, aberrant APP processing, or Aβ synthesis (84,85). Isoflurane, however not desflurane, triggers an increase in the levels of Aβ40 in human CSF 24 h following surgery. In addition, desflurane, however not isoflurane, was associated with a decrease in the levels of Aβ42 at 2 h following surgery under anesthesia (77). Notably, although desflurane alone cannot increase neuronal cell death, it can increase the vulnerability of primary cultured neurons to intracellular and extracellular Aβ1–42 (86). A 6-h exposure to 12% desflurane under marginally hypoxic conditions (18% O_2_) was demonstrated to induce the activation of caspase -3, alter the APP processing, increase the production of Aβ and increase the activity of BACE in H4-APP cells. This effect was partially rescued by the broad caspase inhibitor, benzyloxycarbonyl-VAD, and attenuated by Clioquinol and L-685,458 (84).

The mechanism of action of inhaled anesthetics leading to AD-like neuropathology remains to be elucidated. The data from *in vitro* human models of AD discussed above, if confirmed *in vivo*, may have profound implications in the field of anesthesiology in elderly patients, particularly those already diagnosed with AD (84).

### Upstream mechanisms

The molecular mechanism underlying the progression from anesthesia-exposure to neuronal cell death has been extensively investigated for isoflurane (3,5,9,74–76,79,85,87–91), sevoflurane (81,82,92) and desflurane plus hypoxia (84). These previous studies have demonstrated that inhaled anesthetics induce caspase activation and cellular apoptosis, increase BACE levels, affect APP processing, increase the synthesis and accumulation of Aβ and eventually impair learning and memory.

At the cellular level, signals transmitted by extracellular anesthetics may manifest into different types of responses. Firstly, the expression levels of cytokines, including tumor necrosis factor-α (TNFα), may increase in response to inhaled anesthetics, leading to the inflammation of neurons. It has been previously reported that 2.1–3% sevoflurane in combination with 60% oxygen induces neuroinflammation in the brain of transgenic AD mice by increasing the expression levels of TNFα (71).

Secondly, isoflurane elevates cytosolic calcium levels and since calcium is an important secondary messenger, alterations in its homeostasis is sensed as a danger signal by the cell, leading to the activation of apoptotic pathways, followed by neuronal cell death. Previous studies have provided evidence in favor of this mechanism (92,93). A previous study demonstrated that the inositol trisphosphate receptor (IP3R) antagonist, 2-APB, attenuated the sevoflurane-induced activation of caspase-3 and the accumulation of Aβ in naïve neonatal mice, suggesting that sevoflurane may act through IP3R to affect calcium homeostasis (71). Other previous studies have revealed that isoflurane induces the activation of caspase-3 and the accumulation of Aβ by increasing cytosolic calcium levels, which are regulated by memantine, a partial antagonist of the N-methyl-D-aspartate receptor (NMDAR) (94). A previous study demonstrated that 4 *μ*M memantine inhibits the increase in cytosolic calcium levels, attenuates the activation of caspase-3 and apoptosis, and improves cell viability in response to isoflurane exposure (94). In naïve mice, 20 mg/kg memantine administered intraperitoneally, reduced the isoflurane-induced activation of caspase-3 in the brain (94).

The third possible mechanism of action is through the synthesis of reactive oxygen species (ROS) and the induction of mitochondrial damage. A previous study demonstrated that isoflurane increases the generation of ROS, subsequently causing mitochondrial damage by opening the mitochondrial permeability transition pore, reducing the mitochondrial membrane potential and decreasing adenosine triphosphate levels. This sequence of events leads to the activation of the apoptotic pathways, eventually causing learning and memory impairment (95). The results also demonstrated that cyclosporine A, an inhibitor of the mitochondrial permeability transition pore, inhibits isoflurane-induced opening of the pore *in vitro* and rescues isoflurane-induced deficiencies in learning and memory in mice (95). By contrast, desflurane revealed no mitochondrial damage or caspase activation in the mouse brain and primary neuronal cultures. No impaired learning and memory was observed in these animals (95). Evidence for the affect of sevoflurane and isoflurane on mitochondrial membrane permeability and caspase-3 activation comes from previous studies revealing their apoptotic effect on T lymphocytes. This mechanism was identified to be independent of death receptor signaling. By contrast, desflurane exerted no pro-apoptotic effects (85).

Finally, genetic factors appear to affect the outcome of exposure to inhaled anesthetics. A previous study demonstrated that the cytotoxicity of desflurane was caused by the reduction in miR-214, which normally binds to the 3′ untranslated region of the pro-apoptotic gene, Bax, and represses the expression of this protein. Downregulation of miR-214, therefore, leads to increased expression of Bax and consequently increases neuronal cytotoxicity by the accumulation of Aβ (86). Other previous studies demonstrated that a 2 h exposure to sevoflurane causes the accumulation of Aβ in the brain and exacerbates Alzheimer’s-like pathology by reducing the levels of low-density lipoprotein receptor-related protein 1, increasing the expression of receptor for advanced glycation end products and decreasing the expression levels of IDE and neprilysin in aged and, to a lesser extent, young rat brain (82). Successful downregulation of BACE, full-length APP and APP c-terminal fragments by treatment with small interfering RNAs targeting these transcripts, attenuates the synthesis and accumulation of Aβ, and isoflurane-induced activation of caspase-3 (78).

Isoflurane also increases the ratio of Bax/Bcl-2. A 6 h exposure to 2% isoflurane increases the mRNA expression of Bax and decreases the expression of the anti-apoptotic factor Bcl-2. This increases the accumulation of ROS, facilitates cytochrome *c* release from the mitochondria to the cytosol, induces the activation of caspase-9 and caspase-3, and leads to apoptotic cell death (96). This effect can be attenuated by administration of the intracellular calcium chelator, BAPTA (96). Isoflurane, therefore, appears to induce apoptosis by altering the Bax/Bcl-2 ratio and triggering mitochondrial damage through the generation of ROS (96). This previous study also confirmed that desflurane does not activate the ROS-mediated mitochondrial pathway of apoptosis (96).

These findings have partially demonstrated the upstream mechanisms leading from exposure to isoflurane and sevoflurane to Aβ generation, caspase activation and apoptosis. They have also provided evidence to facilitate future studies aimed at elucidating the mechanism underlying the effect of inhaled anesthetics on the neuropathogenesis of AD.

## 5. Effects of inhaled anesthetics on tau pathology

### Isoflurane

Several previous studies have confirmed that isoflurane can trigger the hyperphosphorylation of tau *in vivo* (97,98). Repeated normothermic exposure to isoflurane significantly increased hippocampal phosphorylation of tau at the AT180 (pTau213/235) epitope in 3 × Tg-AD mice (97). Dong *et al* (98) demonstrated that a 2 h exposure to 1.4% isoflurane increased the phosphorylation of tau at serine 262 (Tau-PS262) in AD-transgenic mice, up to 24 h following administration of anesthesia. An *in vitro* study revealed that primary neuronal cultures derived from AD-transgenic mice exhibited an increase in the expression of Tau-PS262 following a 6 h exposure to 2% isoflurane (98). At clinically relevant doses, a previous study revealed that increased levels of phosphorylated-tau was distributed in the neuropil and cell bodies, increased the levels of insoluble and aggregated forms of tau, and the detachment of tau from microtubules (4). The increase in insoluble tau at 1 week following anesthesia suggested that anesthetics cause molecular changes in the brain, which trigger later the development of tauopathy (4). However, as discussed earlier, a previous study revealed contradictory results, suggesting that isoflurane was not identified to exhibit a significant affect on the levels of tau in human CSF (77).

### Sevoflurane

Sevoflurane can exhibit identical physiological effects to isoflurane. At least two independent previous studies have established a correlation between the exposure to sevoflurane and persistent postoperative cognitive decline, particularly in patients >65 years of age (6,7). In one study, acute exposure to 1.5% sevoflurane was demonstrated to cause a significant, dose-dependent and reversible hyperphosphorylation of tau in the hippocampus of 5–6 month-old C57Bl6/J mice (6). These findings revealed that repeated exposure to 2.5% sevoflurane under normothermic conditions leads to persistent hyperphosphorylation of tau at the Ser396/Ser404 and Thr181 phosphoepitopes. The mice subsequently developed significant deficiencies in spatial learning and memory, as assayed using the MWM test (6). Since hyperphosphorylated tau is a major constituent of neurofibrillary lesions, these previous studies suggest a possible mechanism by which anesthetics may cause postoperative cognitive impairment and increase the risk of AD (7). Nevertheless, a causal association between the burden of phosphorylated tau and cognitive decline remains to be elucidated.

### Desflurane

Desflurane has not been indicated to significantly affect the levels of tau in human CSF between 2 and 24 h following surgery under spinal anesthesia (77). However, whether desflurane can directly or indirectly induce the hyperphosphorylation of tau *in vivo* remains to be elucidated.

### Role of anesthesia-induced hypothermia

Previous studies have demonstrated that anesthesia-induced hypothermia directly induces aberrant hyperphosphorylation of tau (99,100). At a temperature <37°C, an 80% increase in the phosphorylation of tau is observed with each degree of decline in temperature, an effect that was rescued *in vivo* by preventing hypothermia (100). Planel *et al* (99) demonstrated that restoration of core body temperature to normal reversed the increase in the hyperphosphorylation of tau caused not by anesthesia *per se*, but by anesthesia-induced hypothermia. Mechanistically, this type of phosphorylation was not due to the activation of tau kinase, but rather a secondary consequence of the direct inhibition of PP-2A activity by the hypothermic conditions (99,100). Other previous studies have confirmed these findings in different *in vivo* models. For example, Tan *et al* (8) demonstrated that exposure of Sprague-Dawley rats to 1.5% isoflurane without temperature control inhibited the activity of PP-2A and increased the hyperphosphorylation of tau at the Thr-205 and Ser-396 epitopes in the hippocampus. This was, in turn, associated with the deficits in spatial learning and memory observed in hypothermic rats (8). In humans, body temperature is suggested to be a risk factor for AD and hypothermia (common in the elderly) and is hypothesized to increase the pathology of AD (101). However, the impairment in learning and memory in response to isoflurane with temperature control is not accompanied by changes in the expression levels of the total tau protein or phosphorylated tau at Thr231 and Ser396 (102). Further studies are required to ascertain the direct, causal association between anesthesia-induced hypothermia and memory impairment.

### Upstream mechanisms

Le Freche *et al* (6) demonstrated that the activation of AKT, ERK and GSK-3 signaling in response to anesthesia contributes to its pathological consequences. Specifically, this study revealed that normothermic sevoflurane exposure can lead to the dysregulation of the MAPK and AKT/GSK3 pathways (6). As discussed, aberrant hyperphosphorylation of tau due to anesthesia-induced hypothermia and hypothermia-induced hyperphosphorylation of tau results from the direct inhibition of PP-2A activity by the hypothermic conditions rather than the activation of tau kinase (99,100). A study using the NMDAR antagonist, memantine, in a mouse model of tauopathy suggested that NMDAR-mediated signaling may be important in the development of the pathology of tau following isoflurane-induced hypothermia (103). However, other receptors may also contribute to this phenomenon. The mechanism by which memantine antagonizes the deleterious effects of isoflurane leading to tau pathology remains to be elucidated and warrants further investigation.

## 6. Inhaled anesthesia, Aβ, tau hyperphosphorylation and AD

Dong *et al* (98) first reported that clinically relevant exposure to isoflurane increased the expression levels of phosphorylated tau, most likely due to anesthetic-induced caspase activation and generation of Aβ aggregates. These findings demonstrated that the increase in Tau-PS262 levels in brain tissues and primary neurons derived from AD-transgenic mice [B6.Cg-Tg (APPswe, PSEN1dE9)85Dbo/J] following isoflurane exposure is attenuated by Z-VAD and L-685,458 (98). An animal study demonstrated that a combination of 70% nitrous oxide and 1.2% isoflurane is associated with a long-term deficit in learning and memory in young and aged rats (87). Notably, in a previous study on human subjects, desflurane, in contrast to isoflurane, was not observed to induce a decline in cognitive function (104). There are also studies that have suggested that isoflurane may impair learning and memory independent of the accumulation of Aβ, suggesting an alternative pathway upstream of neurodegeneration (105). Other previous studies have revealed that isoflurane, however not desflurane, induces the activation of caspase and the synthesis of Aβ (76,84,96). Notably, AD-like pathology has been demonstrated to recover over time *in vivo*. In 85Dbo/J AD-transgenic mice (APPswe, PSEN1dE9), the Aβ plaques and oligomers observed in the hippocampus decreased significantly during the 5 months following isoflurane exposure (80). Further studies are required to elucidate the association between inhaled anesthetics, Aβ accumulation, tau hyperphosphorylation and the onset of AD.

## 7. Future studies

To date, the effects of isoflurane, sevoflurane and desflurane on Aβ aggregation, tau hyperphosphorylation and cognitive function have been determined. However, several of these independent conclusions are inconsistent and often contradictory. Resolving these discrepancies is required by specifically addressing the following issues: i) The cause-effect association between inhaled anesthetics and Aβ accumulation, tau hyperphosphorylation and neurobehavioral deficits. ii) The effect of anesthetics on the expression levels and activity of the enzymes involved in Aβ degradation (IDE and neprilysin), which may aid in clarifying whether Aβ accumulation is the result of increased generation and/or decreased degradation of Aβ peptides. iii) The upstream mechanisms by which inhaled anesthetics induce caspase activation and cause mitochondrial damage. iv) Comparative analysis of the effects of commonly used inhaled anesthetics, including isoflurane, sevoflurane and desflurane, on the neuropathogenesis and cognitive function of AD in order to identify those with the least deleterious effects. v) Contrasting mechanism of action of different anesthetics, for example, isoflurane versus desflurane, on caspase activation and Aβ accumulation, which may lead to targeted approaches to prevent or treat anesthesia-induced neurotoxicity. vi) It is clear that cognitive decline following major surgery is associated with gliosis, Aβ accumulation and tau phosphorylation in aged mice (106). Since anesthesia also increases the accumulation of Aβ, it is essential to determine possible synergistic effects of anesthetics, including isoflurane, together with surgical procedures, which may lead to more severe cognitive dysfunction. vii) Although there is preclinical evidence supporting the ability of inhaled anesthetics to exacerbate the pathology of tau in a dose-dependent manner, further studies are required to investigate the impact of prolonged or repeated anesthesia exposure in humans, particularly those diagnosed with or at risk of developing AD. viii) Mechanisms by which tau hyperphosphorylation and aggregation leads to synaptic dysfunction and neurotoxicity, and the contribution of anesthetics to this phenomenon. ix) A direct causal link between increased hyperphosphorylation of tau and impaired cognition remains to be elucidated.

## 8. Conclusion

The present review has summarized previous findings on the effects of the commonly used inhaled anesthetics, isoflurane, sevoflurane and desflurane, on the accumulation of Aβ peptides, tau hyperphosphorylation and other AD-like pathologies, *in vitro* and *in vivo*. It is clear that isoflurane and sevoflurane can induce pro-apoptotic signaling, including caspase activation, and cause aberrant APP processing, increased synthesis and accumulation of Aβ, and hyperphosphorylation of tau in cell lines, primary neurons and *in vivo* in the brain. Findings on the effect of desflurane on the generation of Aβ and hyperphosphorylation of tau are contradictory. Several mechanisms have been suggested to explain the role of inhaled anesthetics in inducing caspase activity, Aβ generation, tau hyperphosphorylation and cognitive impairment. These include disruption of calcium homeostasis, mitochondrial damage and downregulation of miR-214. Although previous studies have revealed the deleterious effects of inhaled anesthetics on patients with AD, further studies are required to fully elucidate anesthesia-induced neurotoxicity. Further investigation may assist in the development of future guidelines for the safe administration of anesthetics to patients with AD to avoid worsening cognitive dysfunction.

## Figures and Tables

**Table I:** Currently known common Alzheimer’s disease-associated genes.

Chromosome	Protein	Function	Gene defect	Phenotype
21q21.2	APP	Aβ generation	APP mutation	Inc. production of all Aβ peptides or Aβ40 peptide
14q24.3	PSEN1	Aβ generation	PSEN1 mutation	Inc. production of Aβ42 peptide
1q31-q42	PSEN2	Aβ generation	PSEN2 mutation	Inc. production of Aβ42 peptide
10q.23.33	IDE	Aβ degradation	IDE mutation	Dec. degradation of Aβ peptides
19q13.2	APOE	Aβ clearance/export, Aβ oligomerization	APOE-4 polymorphism	Dec. density of Aβ plaques and vascular deposits

Inc., increased; Dec., decreased; APP, amyloid precursor protein; Aβ, β-amyloid; PSEN, presenilin; IDE, insuilin-degrading enzyme; APOE, apolipoprotein E.
